# Negative trials in ovarian cancer: is there such a thing as too much optimism?

**DOI:** 10.3332/ecancer.2016.ed58

**Published:** 2016-08-17

**Authors:** Bishal Gyawali, Vinay Prasad

**Affiliations:** 1 Department of Hemato-Oncology, Nobel Hospital, Sinamangal, Kathmandu 21034, Nepal; 2 Division of Hematology Oncology, Knight Cancer Institute, Oregon Health and Science University, USA; 3 Department of Public Health and Preventive Medicine, Oregon Health and Science University, 3181 SW Sam Jackson Park Road, Portland, Oregon, USA; 4 Center for Health Care Ethics, Oregon Health and Science University, Portland, Oregon 97239, USA

**Keywords:** ovarian cancer, statistical significance, clinical trials, angiogenesis inhibitor, nintedanib, volasertib

## Abstract

Recently, two clinical trials of novel agents in metastatic ovarian cancer were published: a phase 3 study of nintedanib and a phase 2 study of volasertib. There seemed to be discordance between the results and conclusions in the publication of both these trials. Despite not very optimistic results, the studies concluded optimistically in favor of the new agents under study. Using these examples, we point out the discrepancies and the risks of concluding optimistically based on statistical significance when the actual benefit is minimal. We also appeal against conducting large phase 3 trials that require significant resources without good phase 2 evidence for doing so.

Over the last decade, many large phase III trials have been conducted in an attempt to improve the quantity and quality of life for patients with advanced ovarian cancer. Ovarian cancer remains both common and lethal [1], with most women undergoing treatment with carboplatin and paclitaxel after maximal debulking surgery [2]. Several new drugs have been tried in the first-line and relapsed setting—both for platinum-sensitive and platinum-resistant cases and both as upfront and maintenance strategies—but hardly any meaningful gains in survival have been achieved. The greatest optimism has been for the angiogenesis inhibitors: bevacizumab [3–6], pazopanib [7], trebananib [8] and cediranib [9]. All have been tested but failed to show any benefit in overall survival despite modest gains in PFS for some drugs in some settings (OS data for cediranib aren’t mature yet). Thus, despite years of research, we are yet to add to carboplatin-paclitaxel any drug with proven survival benefit.

One may wonder why so many trials were needed to teach us the same thing: angiogenesis inhibitors do not prolong survival in ovarian cancer. Recently, the ICON-7 trial’s survival results showed an OS benefit with bevacizumab among high-risk patients [3]. Unfortunately this was not the primary study population, but a subgroup. Worse, this study was not placebo-controlled, and notably survival was not prolonged in the entire group [10]. Concern with excess reliance on subgroup analysis [11] is captured in the adage: “if you torture your data enough, it will confess to anything”.

Two recent trials exemplify the problem of excessive optimism in the ovarian cancer trial agenda: the AGO-OVAR 12 [12] and the GINECO-volasertib study [13].

AGO-OVAR 12 was a big phase III study recruiting 1366 chemo naïve ovarian cancer patients, and randomizing them 2:1 to chemotherapy with nintedanib or placebo [12]. The authors ultimately conclude that, “Nintedanib in combination with carboplatin and paclitaxel significantly increases progression-free survival (PFS)”. Here, significant is used to denote *statistical significance*, i.e. that the probability that a result at least this extreme is due to chance falls within our pre-specified alpha error, rather than *clinical significance*, i.e. the benefit in progression free survival has some value to patients.

In fact, the median PFS in nintedanib was 17.2 months vs 16.6 months in placebo arm- a mere 0.6 months benefit. Progression free survival (not overall survival) was prolonged by nearly 18 days on average when nintedanib is added to usual chemotherapy! Is that meaningful to patients? How about when we consider that it must be balanced against a 44% higher risk of grade 2 or more diarrheas? And, we haven’t even mentioned the price, which is certain to be lofty as is with all modern targeted therapies. With nintedanib, we find another example of an overpowered cancer trial: powered to detect a statistical difference with marginal or no clinical value [14].

In addition to being over-powered, the trial’s rationale may be questioned. The study cites a previously conducted phase II study as basis for undertaking this huge phase III trial [15]. Unfortunately, the phase II study used as a basis for this phase III trial had failed to reach “significance” in both the endpoints of PFS (PFS rate at 36 weeks: 16.3% v 5%, HR 0.65, 95% CI 0.41-1.02, p = 0.06) and OS (HR 0.84, 95% CI 0.51-1.39; P=0.51). Also, the PFS rate in both the arms was dramatically low compared to the rates assumed during sample size calculation, ( 70% in nintedanib and 50% in placebo groups ), and the median PFS was indistinguishable between arms. And yet the authors of the phase II study concluded “the results are sufficient to justify a phase III trial”. If a 0.6 months benefit be called “significant” based solely on a p-value, one wonders why this “lack of significance” didn’t prompt the investigators to conclude “phase III trial is not justified” instead. One must wonder about the broader perverse incentives of our system, where even the most marginal drugs can approach billion dollar market shares, prompting companies to continue testing them. Ultimately, the drug-manufacturer made the decision to move this disappointing agent forward, and they did so knowing that any gains in PFS would be trivial—from a business standpoint such a decision only makes sense if the potential reward is tremendous.

In AGO-OVAR 12, no meaningful lessons were learned despite the time and effort of 1300 patients —this huge number of patients could have otherwise been used to address more pressing questions. Of course, the trial was not futile because it was negative; instead, it was futile because it was overpowered (used more patients than needed), and tested a hypothesis that was unlikely to succeed at the outset. Furthermore, although the authors continue to believe that “Further studies of nintedanib are needed…”, we would argue that no further studies are needed. A benefit of 18 days demands no further trials. Knowing when to give up is as important as knowing when to persevere.

Almost simultaneously, a phase II trial comparing volasertib (a selective cell-cycle kinase inhibitor) with investigator’s choice of single-agent chemotherapy for platinum-resistant or –refractory ovarian cancer patients was published [13]. This trial had the primary endpoint of 24-week disease control rate (DCR). This study’s main finding was that the 24-week DCR was 30.6% in the volasertib arm versus 43.1% in the chemotherapy arm (the DCR was lower in volasertib arm). Median PFS was 13.1 weeks in volasertib and 20.6 weeks in chemotherapy arm. Response rate was 13% v 14.5%. What then did the authors conclude? “Single-agent volasertib showed antitumor activity in patients with ovarian cancer”! Volasertib efficacy was lower compared to chemotherapy, irrespective of whether you consider the primary endpoint of 24-week DCR or the secondary endpoints of response rate or PFS. Moreover, the data the investigators chose to highlight-both in the abstract, the PFS curves and the discussion- was: 6 patients were progression free in volasertib at 1 year versus none in the chemotherapy arm. Emphasizing this casual post-hoc observation makes no sense whatsoever except when seen as a desperate attempt to find something to hail the drug as a good one. Our fear with the volasertib spin is that it could lead to another similar phase III trial with large number of patients to say the same thing again: volasertib is not effective in advanced ovarian cancer.

## Conclusion

As a profession, we must learn to call a spade a spade. Ineffective drugs must be acknowledged as such by the oncology community. Additionally, conducting large phase III trials entails a huge sum of financial, logistic and human resources which is not worth spending on questions that are foolish, ill-supported or over-powered at the outset. Care must be made to clarify if results are statistically or clinically significant, and above all caution should always be observed during the interpretation of clinical trial data. The true lessons of negative trials in ovarian cancer apply broadly to all fields of oncology.

## Conflicts of Interest

None.
